# Effective management of continuous salivary flow through a pharyngocutaneous fistula using a negative pressure wound therapy device

**DOI:** 10.1016/j.jpra.2024.10.016

**Published:** 2024-11-01

**Authors:** Taku Maeda, Nayuta Tsushima, Kosuke Ishikawa, Yuhei Yamamoto

**Affiliations:** a Department of Plastic and Reconstructive Surgery, Faculty of Medicine and Graduate School of Medicine, Hokkaido University, Kita-15 Nishi-7, Kita-ku, Sapporo City, Hokkaido 060-8638, Japan; b Department of Otolaryngology-Head and Neck Surgery, Faculty of Medicine and Graduate School of Medicine, Hokkaido University, Kita 15, Nishi 7, Kita-ku, Sapporo City, Hokkaido 060-8638, Japan

**Keywords:** Esophagojejunal anastomotic fistula, Free jejunal transfer, Negative pressure wound therapy, Saliva, Slit drain

## Abstract

Esophagojejunal anastomotic fistula is difficult to treat because of continuous salivary flow. This report describes the innovative use of a negative pressure wound therapy device with a slit drain to treat an esophagojejunal anastomotic fistula after free jejunal transfer. Insertion of a slit drain was very effective for management of saliva. This device worked as a portable suction machine, allowing earlier healing without the need for surgery. A negative pressure wound therapy device using a slit drain can be useful for recalcitrant esophagojejunal anastomotic fistula with salivary flow after free jejunal transfer.

## Introduction

Free jejunal transfer has become a standard treatment option after total pharyngolaryngectomy with a reported success rate of >95 %, allowing maintenance of oral secretions and a rapid return to oral feeding and swallowing.^1^ Because free jejunal transfer can fail as a result of relatively few complications compared with other reconstructive methods, the rate of fistula formation is high. Furthermore, this type of fistula is very difficult to treat, particularly when it occurs close to anastomosis of the esophagus and the transferred jejunum and involves salivary flow.

Negative pressure wound therapy (NPWT) was first used for management of recalcitrant wounds by Argenta and Morykwas in 1993^2^ and can now be used to treat wounds in the head and neck. A recent review indicated that NPWT is an effective treatment for head and neck wounds and should be considered for patients in whom wound healing is likely to be difficult, including those with a history of head and neck cancer, oro/pharyngocutaneous fistula, and trauma.^3^

This report describes a patient with an esophagojejunal anastomotic fistula with continuous salivary flow after free jejunal transfer that healed with treatment using a negative pressure wound therapy device with a slit drain.

## Case report

A 62-year-old man with a history of hoarseness and pharyngeal pain presented to the Department of Otorhinolaryngology at our hospital. He had a Brinkman index of 800 and a body mass index of 15.79. He had no history of radiotherapy or chemotherapy. Nasopharyngolaryngoscopy detected a nodule at the supraglottis, which was diagnosed as squamous cell carcinoma on biopsy. At the time of surgery, the mass was found to be enlarged and causing dyspnea. Therefore, tracheostomy was performed. Several days later, we performed total pharyngolaryngoesophagectomy, bilateral lymph node dissection, and reconstruction by free jejunal transfer. The right lingual artery and the right internal jugular vein were used as the recipient vessels. The caudal margin of the esophagus was adjacent to the permanent tracheostoma. An esophagojejunal anastomosis was performed using the Gambee suture technique. Color Doppler ultrasonography was used intraoperatively to confirm the viability of the transferred jejunum.^4^

**Figure 1:**
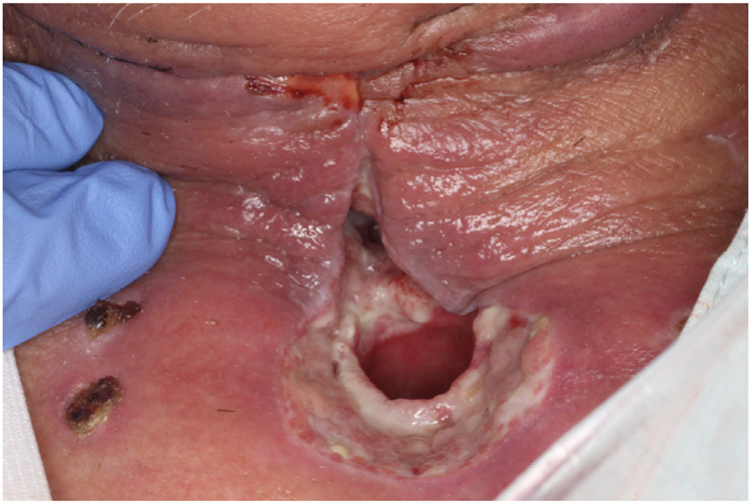
A fistula formed between the skin and the transferred jejunal flap at the site of the tracheostoma.

**Figure 2:**
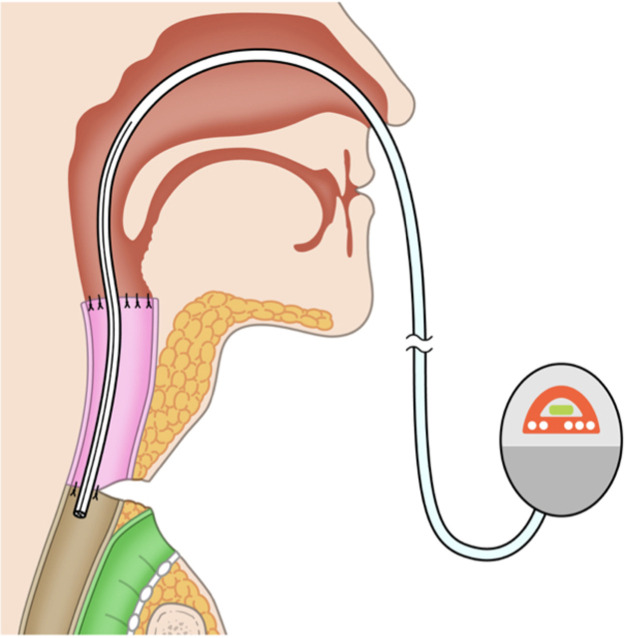
Schema of Negative pressure wound therapy device using a drain was set to aspirate saliva, with the tip of the drain placed beside the fistula.

**Figure 3:**
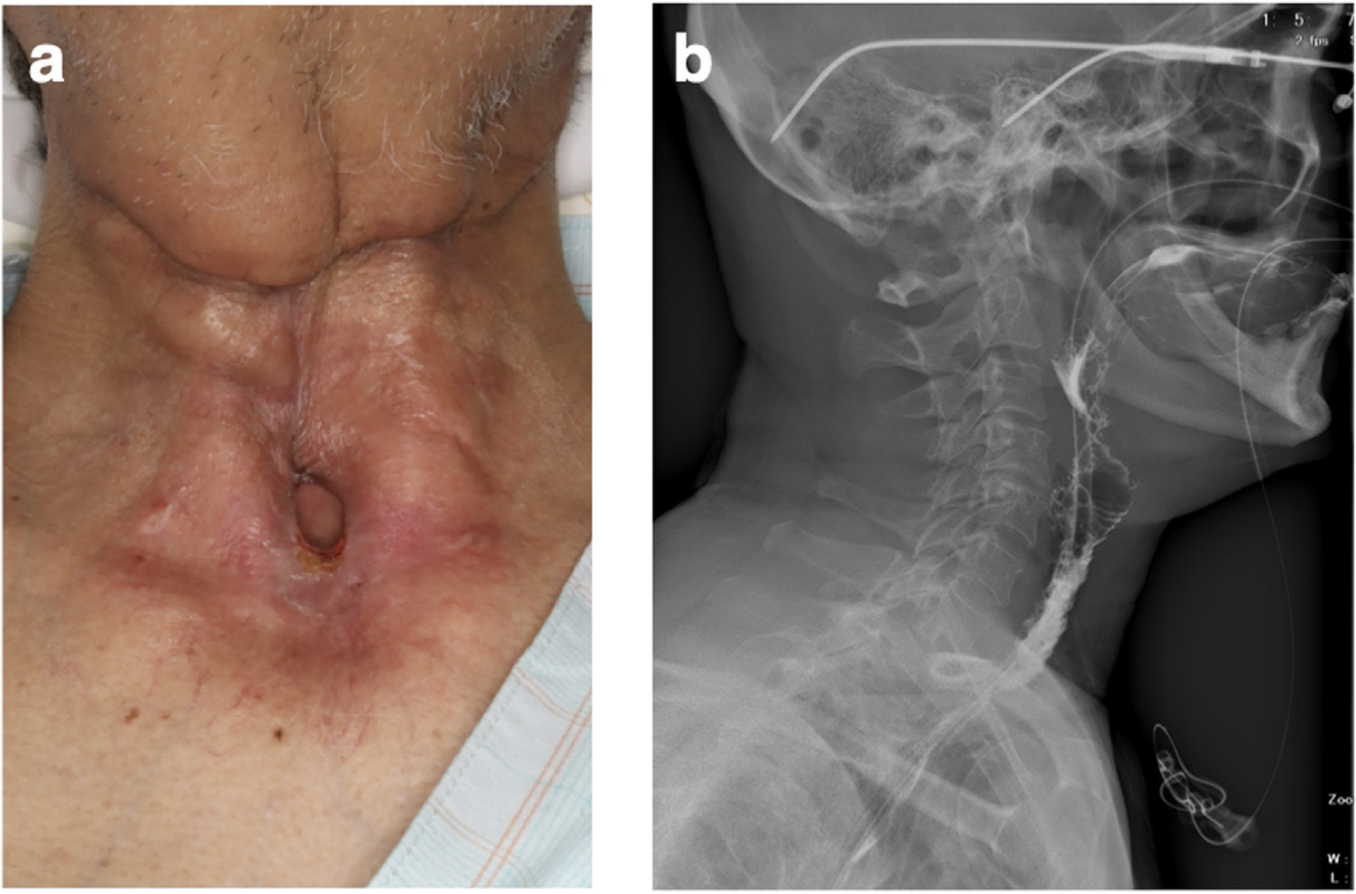
(a) The wound was healed after 37 days of treatment. (b) Swallowing videofluorography confirmed no leak.

Postoperatively, the jejunum survived without thrombosis in the vessel. However, on postoperative day 16, we noted that a fistula had formed between the skin and the transferred jejunum around the tracheostoma (Figure 1). Saliva was flowing continuously through the fistula. Conservative management with gauze was unsuccessful. The fistula had formed between the sutured area of skin on the neck and the posterior wall of the tracheal foramen and passed through the transferred jejunal flap, which was not amenable to NPWT. Conventional NPWT could not applied for this case because the fistula at the site of the esophagojejunal anastomosis was very close to the permanent tracheostoma and it was impossible to maintain stable negative pressure. Therefore, treatment to continuously and effectively aspirate saliva was started using a RENASYS TOUCH device (Smith & Nephew, Watford, UK) (Figure 2). A slit drain was used and inserted through the nasal cavity, pharynx, free jejunal flap, and cervical esophagus because it had a long groove on one side that allowed efficient aspiration of saliva. Thereafter, the wound gradually healed without infection. After 21 days, the fistula was closed, and after 37 days, the wound was healed (Figure 3a). Swallowing videofluorography confirmed that there was no leak (Figure 3b). Oral intake was possible, although one endoscopic balloon dilation procedure was necessary. No recurrence of the fistula was noted at 6 months postoperatively.

## Discussion

This case highlights two important clinical points. First, a simple negative pressure would therapy device using a slit drain is a useful strategy for a recalcitrant esophagojejunal anastomotic fistula with continuous salivary flow after free jejunal transfer. Second, patients can participate in a rehabilitation program during this treatment, which promotes more rapid wound healing.

In regard to the first point, NPWT has been reported to be an excellent alternative for management of complicated wounds after head and neck reconstruction. This method is safe and comfortable for the patient and provides good results in terms of infection control, obliteration of the dead space, and improvement of wound healing.^5^ Complications such as fistula are rare but devastating for the patient, prolong the hospital stay, and impede rehabilitation. Therefore, early resolution of any such complications is important. In recent years, NPWT has been used to treat complicated wounds with fistula. However, the following two conditions have to be met in order to treat a fistula with NPWT. First, there must be an adequate amount of surrounding soft tissue to close the fistula. Second, it must be possible to maintain stable negative pressure without leak of air or saliva.^6^ In the case described here, the fistula at the site of the esophagojejunal anastomosis was very close to the permanent tracheostoma, so it was impossible to maintain stable negative pressure without leak of air or saliva. Furthermore, the size of the fistula on the skin side was comparatively large, and there was not sufficient surrounding tissue.

Therefore, conventional NPWT was not appropriate in this case. Instead, we focused on management of salivary flow through the fistula. Excessive salivary flow can usually be treated with aggressive wound care, pharmacologic inhibition, radiation, or surgery.^7^ The simplest method for control of saliva is to divert its flow away from important structures. In this case, when wound care using gauze was unsuccessful, a slit drain inserted through the nasal cavity, pharynx, free jejunal flap, and cervical esophagus was implemented to control saliva leakage through the fistula. A slit drain that could take in as much saliva as possible via its side was chosen rather a drain with a drainage end and no slit. The slit drain was connected to a Renasys Touch negative pressure collection device, after which most of the saliva was suctioned and wound healing was accelerated.

The second issue highlighted by the present case is that use of the negative pressure wound therapy device enabled the patient to undertake rehabilitation, which promoted wound healing. The suction device is usually attached to a wall; however, the device used in our patient was small and portable. Therefore, the patient could walk around the ward and undertake daily rehabilitation exercises without restriction while wearing this device. There is some experimental evidence suggesting that lack of physical activity can impair healing of skin wounds.^8^ Furthermore, an adjunctive exercise program has been shown to promote ulcer healing.^9^ For survivors of head and neck cancer, physical exercise interventions have improved physical function, muscle endurance, range of motion, and overall quality of life and alleviated pain and fatigue.^10^ It is recommended that patients undergoing particularly invasive head and neck surgery receive regular rehabilitation therapy to restore their functional activity and not be confined to bed for a lengthy period after surgery. In the past, NPWT required wall suction at the bedside, but the advent of a portable device has allowed patients to mobilize and undertake early rehabilitation. Mobilization under the supervision of a physiotherapist should be as early and intensive as possible. The portable negative pressure device was demonstrated to improve the rate of wound healing.

In conclusion, this case demonstrated that a simple negative pressure wound therapy device with a slit drain was useful for treating a recalcitrant esophagojejunal anastomotic fistula with continuous salivary flow after free jejunal transfer. This method allowed the patient to participate in rehabilitation during treatment and accelerated wound healing.

## Details of any meeting at which the work was presented, wholly or in part

The 47th Annual Meeting of the Japan Society for Head and Neck Cancer.

## Statements and declarations

None.

## Funding

The authors declare that no funds, grants, or other support were received during the preparation of this manuscript.

## Consent to participate

Informed consent was obtained from the patient included in the study.

## Ethical approval

Not required.

## CRediT authorship contribution statement

**Taku Maeda:** Conceptualization, Data curation, Investigation, Writing—original draft. **Nayuta Tsushina and Ishikawa Kosuke:** Writing—review and editing. **Yuhei Yamamoto:** Supervision, Writing—review and editing.

## Declaration of competing interest

The authors have no relevant financial or non-financial interests to disclose.
